# Characterization and Expression of the Cytochrome P450 Genes in *Daphnia magna* Exposed to Cerium Oxide Nanoparticles

**DOI:** 10.3390/ijms251910812

**Published:** 2024-10-08

**Authors:** Xinyi Kang, Yan Zhou, Qi Liu, Miao Liu, Jing Chen, Yuanwen Zhang, Jie Wei, Yuan Wang

**Affiliations:** Key Laboratory of Hydrobiology in Liaoning Province, Dalian Ocean University, Dalian 116021, China; xinyi_kang@foxmail.com (X.K.); yan_zhou1@foxmail.com (Y.Z.); liuqi_67a@foxmail.com (Q.L.); miao.liu6@foxmail.com (M.L.); sleep1115@foxmail.com (J.C.); yuanwen_zhang@foxmail.com (Y.Z.)

**Keywords:** metal oxide nanoparticles, gene family, aquatic invertebrates, detoxification, transcriptome

## Abstract

As cerium oxide nanoparticles (nCeO_2_) continue to infiltrate aquatic environments, the resulting health risks to exposed aquatic organisms are becoming evident. Cytochrome P450 (CYP) enzymes are integral to the detoxification processes in these species. Herein, we conducted a genomic analysis of CYPs in *Daphnia magna*, encompassing phylogenetic relationships, gene structure, and chromosomal localization. We identified twenty-six CYPs in *D. magna*, categorizing them into four clans and seven families, distributed across six chromosomes and one unanchored scaffold. The encoded CYP proteins varied in length from 99 to 585 amino acids, with molecular weights ranging from 11.6 kDa to 66.4 kDa. A quantitative real-time PCR analysis demonstrated a significant upregulation of CYP4C1.4, CYP4C1.5, CYP4C1.6, CYP4c3.3, and CYP4c3.6 in *D. magna* exposed to 150 mg/L nCeO_2_ for 24 h. The transcript levels of CYP4C1.3, CYP18a1, CYP4C1.1, and CYP4c3.9 were notably downregulated in *D. magna* exposed to 10 mg/L nCeO_2_ for 48 h. A further transcriptomic analysis identified differential expression patterns of eight CYP genes, including CYP4C1.3, in response to nCeO_2_ exposure. The differential regulation observed across most of the 26 CYPs highlights their potential role in xenobiotic detoxification in *D. magna*, thereby enhancing our understanding of CYP-mediated toxicological responses to metal nanoparticles in aquatic invertebrates.

## 1. Introduction

Cerium oxide nanoparticles (nCeO_2_) represent a critical class of engineered metal oxide nanomaterials [1]. nCeO_2_ finds extensive applications across diverse sectors such as the energy, chemical, biomedical, and environmental fields [2,3]. Projections indicate that by 2050, the annual production of nCeO_2_ will reach approximately 10,000 tons [4]. Consequently, it is estimated that the technosphere (e.g., landfills, waste disposal) receives up to 4000 tons of nCeO_2_ annually, while the ecosphere (waters, soil, air) receives up to 300 tons [5]. Once unleashed into aquatic environments, nCeO_2_ may exhibit potential toxicity to a wide diversity of organisms, including various invertebrates [6]. Research has demonstrated that nCeO_2_ exhibits both acute and chronic toxicity to a spectrum of aquatic organisms, including the cladoceran *Daphnia magna* [5], freshwater bivalve *Dreissena polymorpha* [7], rainbow trout *Oncorhynchus mykiss* [8], zebrafish *Danio rerio* [9], and microcrustacean *Chydorus sphaericus* [6]. nCeO_2_ may induce harmful effects across various trophic levels, including modifications in brain enzyme activity, genotoxicity, embryotoxicity, and histopathological damage to target organs in aquatic organisms such as the goldfish *Carassius auratus* [10], microalga *Pseudokirchneriella subcapitata* [11], *D. rerio* [6], planarian *Dugesia japonica* [12], and *O. mykiss* [8].

The primary adverse physiological effects observed in aquatic organisms [13] exposed to nCeO_2_ include detrimental impacts on fitness-related traits such as reproduction, immunity [14], swimming performance [7], growth, and development [7,15]. Owing to its redox properties, which facilitate the transition between Ce^3+^ and Ce^4+^ oxidation states, nCeO_2_ can function as either an antioxidant or a reactive oxygen species (ROS) producer within cells [1,7]. nCeO_2_ is capable of inducing ROS generation [15,16], apoptosis [17], autophagy, and inflammation [18] at the cellular level. Even at low levels, nCeO_2_-induced increases in ROS under oxidative stress conditions can result in premature cell cycle interruption or differentiation in progenitor cells. This leads to ROS-induced apoptosis, which in turn causes abnormal development during zebrafish embryogenesis [19]. Previous research demonstrated that exposure to nCeO_2_ disrupted antioxidant capacity at the molecular level, increased DNA damage, and reduced stem cell proliferation in *D. japonica* [12]. Transcriptomic analysis of the microalga *Chlamydomonas reinhardtii* exposed to nCeO_2_ revealed the identification of Ce ion-specific genes (Cre17g.737300, MMP6, GTR12, and HSP22E) and two oxidative stress biomarkers (APX1 and GPX5) associated with nCeO_2_ toxicity [20].

The cytochrome P450 (CYP) superfamily comprises heme-containing enzymes with monooxygenase, peroxidase [21], and peroxygenase activities, playing a crucial role in phase I of xenobiotic metabolism [22]. CYPs possess a heme domain and, during a catalytic cycle, utilize a redox protein or domain to facilitate the transfer of electrons from NAD(P)H to the heme iron [23]. CYPs catalyze the introduction of oxygen atoms into lipophilic compounds, thereby enhancing their solubility and facilitating excretion [24,25]. These enzymes are capable of metabolizing a variety of substances, including drugs, heavy metals, Bisphenol A (BPA) [26,27], polycyclic aromatic hydrocarbons (PAHs) [28], trichlorfon [29], and benzo[α]pyrene (B[α]P) [22,30], into nontoxic products such as dihydrodiol, phenolic, and epoxide intermediates in aquatic organisms [30]. This detoxification process is essential for safeguarding organisms against the deleterious effects of environmental contaminants. Based on amino acid sequence similarities and phylogenetic relationships [31], CYP genes are categorized hierarchically into clans, families, and subfamilies [29,32,33]. CYPs families 1–3, along with certain members of CYP4 family, are primarily responsible for the detoxification and metabolization of xenobiotics [33], including drugs, heavy metals, and chemical toxicants. CYPs families 5–51 work a vital role in the metabolic processes of endogenous molecules, including steroids and fatty acids [29,34]. Numerous CYP genes have been identified in various aquatic species, including the sea squirt *Ciona robusta* [35], carp *Cyprinus carpio* L. [36], cladoceran *Diaphanosoma celebensis* [22], crayfish *Faxonius virilis* [37], spotted sea bass *Lateolabrax maculatus* [29], and lobster *Panulirus ornatus* [33], and their roles in detoxification have been extensively studied.

Previous studies have demonstrated that CYP genes’ expression in response to various stressors, such as temperature, hypoxia [29], and toxicants [27], underscore the adaptive mechanisms of aquatic organisms. Benzophenones can significantly upregulate CYP3A65 gene expression in zebrafish larvae, potentially mediating the oxidative metabolism of estrogen, thereby increasing DNA damage [38]. Overexpression of stress-responsive genes CYP1A and CYP1B has led to the detoxification and bioactivation of B[α]P in black rockfish (*Sebastes schlegelii*) [30]. BPA regulated CYP1B1 expression by downregulating miR-27b-3p, inducing oxidative stress and apoptosis via the mitochondrial pathway in carp spleen lymphocytes [26]. Significant decreases in zebrafish CYP19a1a expression at 1000 µg/L concentration of BPA reduced oocyte maturation and altered normal oogenesis, demonstrating that CYP19a1a plays a crucial role in estrogen synthesis [27]. CYP18A1 and CYP15A1 can catalyze the epoxidation of methyl farnesoate to juvenile hormone in zebrafish exposed to BPA [33]. In the oyster *Crassostrea brasiliana*, the CYP2AU1 gene is involved in phenanthrene metabolism [39], while novel CYP3A isoforms in the marine mussel *Mytilus coruscus* respond to cadmium and B[α]P exposure [40]. The upregulation of four CYP1 genes, namely CYP1A, CYP1B, CYP1C1, and CYP1C2, in *S. schlegelii* exposed to PAHs underscores the crucial role of these enzymes in responding to toxic compounds [30]. These findings emphasize the importance of CYPs in the adaptive responses of aquatic animals to toxicants.

*D. magna* is a pivotal model organism in ecotoxicological studies owing to its sensitivity to environmental stressors, rapid life cycle, and ease of laboratory culture [41,42]. Previous evaluations of the toxicity effects of metal oxide nanoparticles revealed that ZnO, TiO_2_, CuO, CeO_2_, and SiO_2_ adversely impacted the morphology, development, reproduction, and lifespan of *D. magna* [5,43,44,45]. Recent studies have also explored the cytotoxicity [46] and multigenerational toxicological effects [47] of binary mixtures of silver nanoparticles and glyphosate on *D. magna*, revealing that co-toxicity induces significant biochemical responses indicative of oxidative damage. This damage adversely affects the acclimatization and survival of *D. magna* and negatively impacts the growth and reproduction of offspring [5,48]. To investigate the molecular responses of CYP genes to various xenobiotics, qPCR and RNA-seq analyses were performed on *D. magna*. The findings demonstrated that exposure to chlorpyrifos, a pesticide, and paraquat, a herbicide, markedly upregulated the expression of CYP360A8 [49,50], while sublethal concentrations of benzotriazole elicited a comparable upregulation of CYP4C3 expression [51]. Nevertheless, the roles of CYP genes in mediating tolerance to various metal nanoparticle stresses in aquatic organisms remain understood. Herein, we performed a comprehensive genome-wide identification of the CYP gene family in *D. magna* and further examined their expression profiles following exposure to nCeO_2_.

The objectives of this study were: (1) to characterize CYP genes in the *D. magna* genome based on their sequence conservation, chromosomal location, structure, and phylogenetic relationships, (2) to assess the transcriptional regulation of CYP genes in *D. magna* upon nCeO_2_ exposure through quantitative PCR and transcriptomic data, and (3) to evaluate the involvement of CYPs in the detoxification and stress response pathways of *D. magna*. By characterizing the transcriptional profiles of selected CYP genes, we will attempt to reveal the mechanisms underlying nCeO_2_-induced toxicity and define the genes that may be used as bioindicators for environmental surveillance. This research will help in the identification of the toxic effects of metal oxide nanoparticles in aquatic invertebrates.

## 2. Results

### 2.1. Identification of CYP Genes Family Members in Daphnia magna

We identified CYPs in the *D. magna* genome based on the transcriptomic data and using BLAST and HMM methods of *D. magna* CYPs. The presence of the cytochrome P450 domain (PF00067) was verified in all 26 identified CYP genes using both Pfam and Conserved Domain Database (CDD) approaches (Table 1). The CYP genes molecular function in *D. magna* was shown in Table 1. The molecular function of the 26 CYP genes is similar in each clan: the main function of Clan 2 is steroid hydroxylase activity, Clan 3 and Clan 4 mainly exerts monooxygenase activity and oxidoreductase activity, and Mitochondrial Clan involved in ecdysone biosynthesis process and midgut development. The coding sequence lengths, molecular weights (MWs), subcellular localizations, and isoelectric points (PIs) of these CYP proteins are detailed in Table 2. Among the identified CYP proteins, CYP3A19 was the smallest, comprising only 99 amino acids (aa), while CYP18a1 was the largest with 585 aa. The MWs of these CYP proteins ranged from 11.6 kDa (CYP3A19) to 66.4 kDa (CYP306a1). Among the twenty-six CYP proteins, nine were predicted to localize to the endoplasmic reticulum, ten to the plasma membrane, three to the extracellular space, and four to the mitochondria. The PIs of these CYP proteins ranged from 5.93 (CYP4C1.3) to 9.43 (CYP2C15) (Table 2).

### 2.2. Chromosomal Distribution of CYPs in Daphnia magna

**Figure 1:**
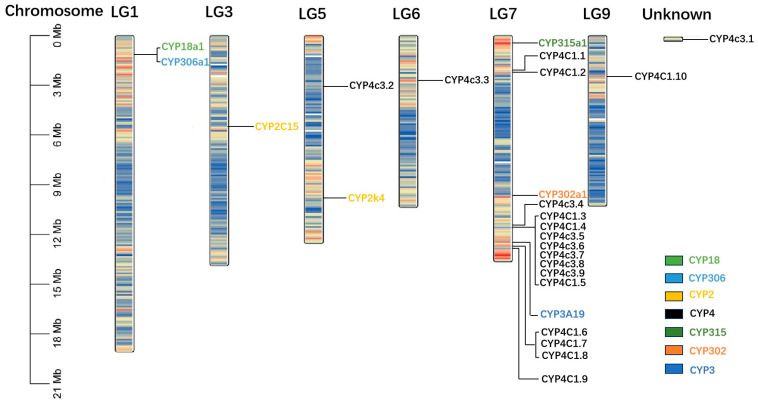
Chromosomal location of the 26 Cytochrome P450s in *Daphnia magna*. The genes with the same color indicate that they belong to the same CYP family based on the phylogenetic tree.

The CYPs of *D. magna* were distributed across six chromosomes and one unanchored scaffold (Figure 1). These CYP genes were unevenly distributed among the seven distinct chromosomes, with each chromosome containing between one and eighteen genes. The highest concentration of CYP genes, totaling sixteen, was found on chromosome LG7. In contrast, chromosomes LG3, LG6, LG9, and the unanchored scaffold each harbored only a single CYP gene. However, some CYP genes from the same family were dispersed across different chromosomes in *D. magna*. For instance, CYP4 family members were distributed across four chromosomes—LG5, LG6, LG7, LG9—and the unanchored scaffold.

### 2.3. Phylogenetic Analysis of CYPs

**Figure 2:**
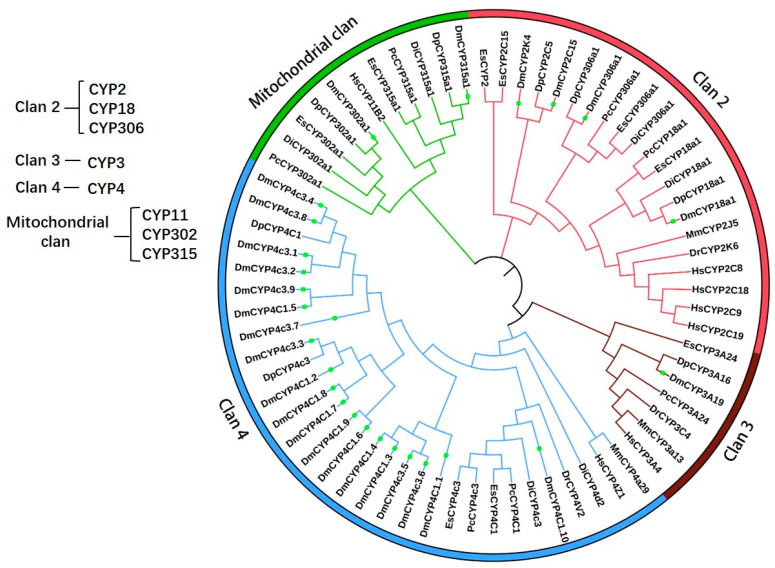
Phylogenetic analysis of Cytochrome P450 proteins in *Daphnia magna*. Clan 2, 3, 4, and Mitochondrial Clan are shown in red, brown, blue, green colors, respectively. *D. magna* CYP proteins are marked by green rectangle.

To elucidate the evolutionary relationships of the identified CYPs in *D. magna* and other species, a circular phylogenetic tree was constructed using MEGA 11 software, depicting the evolutionary history of CYP proteins across species including *D. magna*, *Penaeus chinensis*, *Drosophila innubila*, *Daphnia pulex*, *Danio rerio*, *Mus musculus*, *Homo sapiens*, and *Eriocheir sinensis* (Figure 2). The twenty-six CYP proteins clustered into four distinct clans (Clan 2, Clan 3, Clan 4, and the mitochondrial clan). *D. magna* CYP proteins were predominantly found in Clan 2 and Clan 4, comprising four and nineteen proteins, respectively, with only one protein in Clan 3 and two proteins in the mitochondrial clan. Seven CYP proteins from *D. magna* (CYP3A19, CYP18a1, CYP306a1, CYP2C15, CYP315a1, CYP302a1, and CYP4c3.3) were clustered within a phylogenetic clade alongside homologous CYP proteins from *D. pulex*. The taxonomic relationships based on CYP proteins showed that mammals such as *H. sapiens* and *M. musculus* were clustered into one distinct group, whereas *D. magna* and *D. pulex* formed another.

### 2.4. Motifs and Structures of CYPs in Daphnia magna

**Figure 3:**
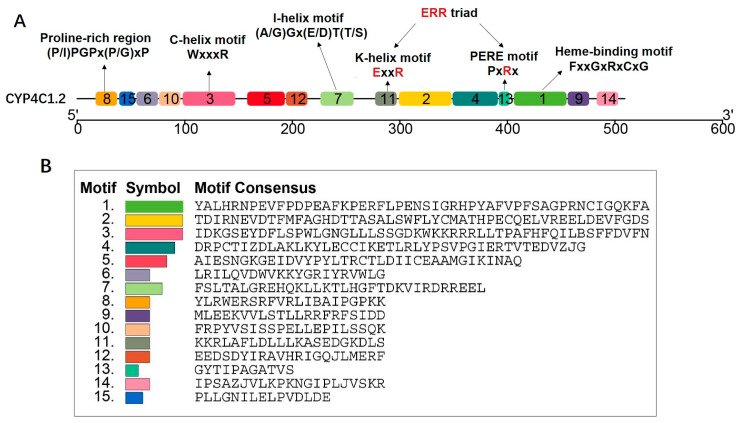

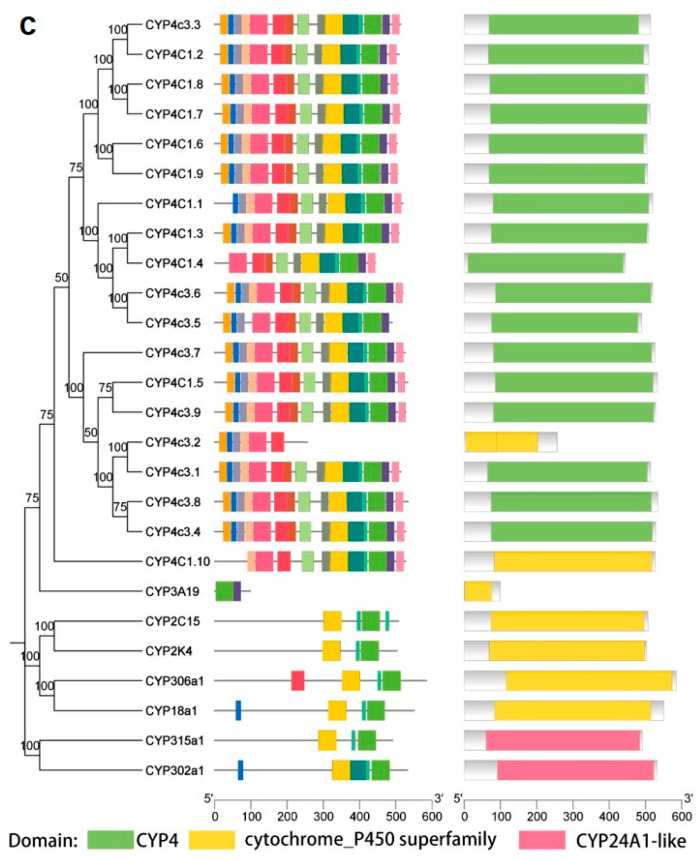
Distribution of conserved motifs and domains within *Daphnia magna* Cytochrome P450s. (**A**) A schematic diagram showcases the conserved motifs within *D. magna*’s CYP proteins, using CYP4C1.2 as a reference. It delineates the signature motifs that encompass functionally significant domains, with the 5′ and 3′ indicating the N-terminal and C-terminal regions. (**B**) Fifteen conserved motif proteins of the CYPs, each small box indicating a motif. (**C**) Distribution of 26 CYPs Phylogenetic tree, 15 conserved motifs, and the domain in *D. magna*.

Fifteen conserved motifs were identified in the *D. magna* CYP proteins through MEME analysis (Figure 3A–C). The majority of these motifs were conserved throughout the *D. magna* CYPs, with four motifs (1, 9, 13, and 14) localized to the C-terminal and another four (6, 8, 10, and 15) situated at the N-terminal. Among the eight conserved motifs, three (motifs 1, 8, and 13) contained functionally characterized domains (Figure 3A). Motif 1 contained the core catalytic center, encompassing the heme-binding motif (FxxGxRxCxG), where cysteine (C) acts as the axial ligand to the thiolate heme. Motif 5 contained the C-helix motif (WxxxR), where the W and R residues contribute to interactions with the propionate side chain of the heme [52]. Motif 6 encompassed the consensus sequence (A/G)Gx(E/D)T(T/S) of the I-helix motif, which is involved in oxygen binding and activation. Motif 8 included a proline-rich region with the consensus sequence (P/I)PGPx(P/G)xP, considered essential as a membrane hinge critical for the proper orientation of CYP enzymes to the membrane. Motifs 11 and 13 featured the K-helix motif (ExxR) and PERF motif (PxRx), respectively (Figure 3A,B).

**Figure 4:**
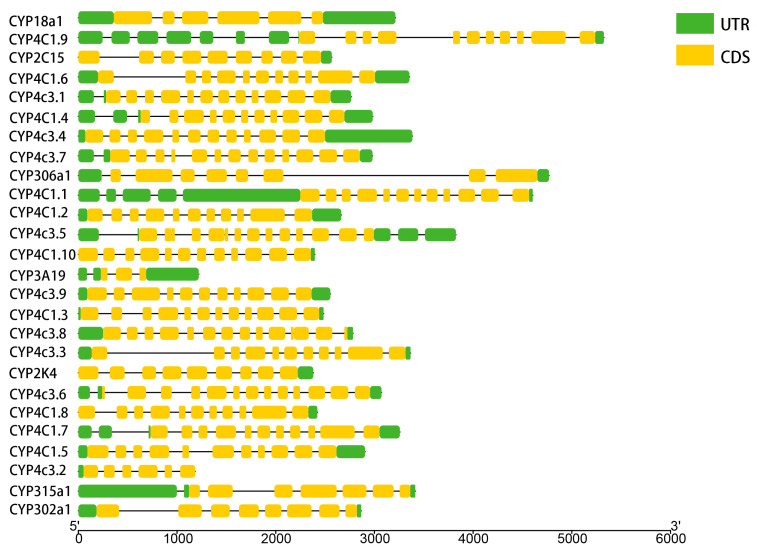
Gene structure analysis of Cytochrome P450s in *Daphnia magna*. The structures of intron and exon and untranslated regions (UTR) are shown with a black line and yellow and green boxes, respectively. The scale is helpful for gene length estimation.

A phylogenetic tree was constructed for the *D. magna* CYP genes, with corresponding motifs and domains displayed adjacent to each gene (Figure 3C). Analysis using MEME motifs revealed that the fewest motifs were observed in proteins from Clan 2, Clan 3, and the Mitochondrial Clan. In Clan 2, only motifs 1, 2, 5, and 13 were identified. Within the Mitochondrial clan, only motifs 1, 2, 4, 13, and 15 were detected. Notably, Clan 3, containing a single member, possessed only motifs 1 and 9. Most CYP proteins within Clan 4 possessed all 15 motifs (Figure 3C). Further domain analysis showed that the majority of CYP genes contained CYP4 and cytochrome P450 superfamily domains (Figure 3C). Only two CYP genes within the Mitochondrial Clan harbored the conserved structural domain CYP24A1-like. Clan 4 exhibited the highest exon count, with CYP4C1.9 containing 18 exons, making it the longest gene among all CYP genes. The exon count within Clan 2 and the Mitochondrial Clan varied between six and nine. Genes within the same clan displayed a similar exon count (Figure 4).

### 2.5. Gene Expression Analysis of CYP Genes in Daphnia magna Exposed to nCeO_2_

**Figure 5:**
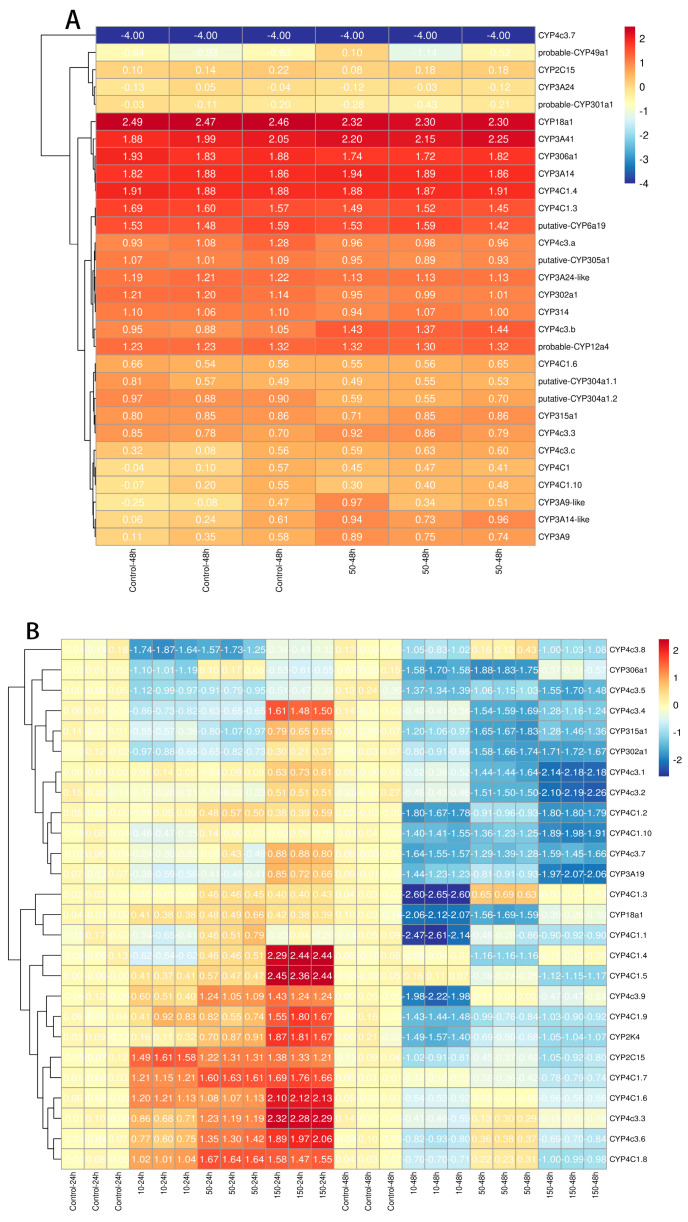
Expression profiles of CYP genes in *Daphnia magna* upon nCeO_2_ exposure. Blue and red colors show low and high relative expression levels, respectively. (**A**) The CYP genes of transcriptomic data in *D. magna* exposed to nCeO_2_ at concentrations of 0 and 50 mg/L at 48 h. (**B**) The CYP gene expression profiles of *D. magna* after 24 and 48 h of exposure to nCeO_2_ (0, 10, 50, and 150 mg/L) were analyzed using qPCR.

We investigated the expression levels of the CYP gene family in response to nCeO_2_ stress in *D. magna*. The transcriptomic data of eight of the eleven genes belonging to the CYP family are consistent with qPCR results (Figure 5A,B). Six of these genes, CYP4C1.4, CYP18a1, CYP302a1, CYP306a1, CYP315a1, and CYP2C15, were downregulated. Two of these genes, CYP4c3.3 and CYP4C1.6, were upregulated. CYP2C15 and CYP4C1.8 were the highest expressed in nCeO_2_ at concentrations of 10 mg/L and 50 mg/L at 24 h, respectively (Figure 5B). CYP4C1.5 had the highest expression in nCeO_2_ at a concentration of 150 mg/L at 24 h; the mRNA level of CYP4C1.5 was 24-, 6-, and 5-times higher than that concentration of 0, 10, and 50 mg/L at 24 h nCeO_2_, respectively. However, as the exposure time progressed to nCeO_2_, most CYP expression levels decreased, and CYP4C1.3 showed the lowest expression in nCeO_2_ at a concentration of 10 mg/L at 48 h, the expression of CYP4C1.3 lower than that concentration of 0, 50, and 150 mg/L at 48 h nCeO_2_ by -26, -4, and 87 times, respectively. Among the 26 genes, CYP4c3.8 had a slight expression in most situations. The expression levels of all CYPs belonging to the mitochondrial clan remained low at both time points and only slightly elevated at a concentration of nCeO_2_ 150 mg/L at 24 h (Figure 5A,B)

## 3. Discussion

Engineered metal oxide nanoparticles, including nCeO_2_, are widely employed across a range of industries and consumer products, leading to their inevitable release into the environment [53]. Once in aquatic ecosystems, nCeO_2_ can interact with and be absorbed by aquatic organisms, potentially leading to toxic effects [54]. Empirical studies have demonstrated that nCeO_2_ induces cellular damage, oxidative stress, and inflammatory responses in aquatic species [8,54]. Elucidating the molecular mechanisms underpinning these effects is pivotal for evaluating the ecological risks associated with metal oxide nanoparticle exposure [55]. CYP enzymes play a key role in the detoxification of harmful substances, including heavy metals, BPA, PAHs, B[α]P, and trichlorfon [26,27,28,29,30], in aquatic species [56]. Investigating the characterization and expression profiles of CYP genes in *D. magna* subjected to nCeO_2_ exposure offers critical insights into the molecular mechanisms underlying nanoparticle-induced toxicity.

We identified 26 CYP genes in the *D. magna* genome using a combination of BLAST search, HMM modeling (PF00067), and NCBI-CDD analysis. This finding is consistent with the CYP gene count observed in other invertebrates, such as the Ornate Spiny Lobster *Panulirus ornatus* [33] and marine rotifer *Brachionus rotundiformis* [25], but is lower than those found in vertebrates like zebrafish *Danio rerio* [34] and black rockfish *Sebastes schlegelii* [30]. The 26 identified CYPs, including CYP2, CYP4, and CYP316, are primarily localized in the endoplasmic reticulum, mitochondria, and Golgi apparatus (Table 2). These CYPs are hydrophilic proteins with GRAVY coefficients below zero, which is consistent with their roles in cellular detoxification and metabolic processes (Table 2). CYP3A19, the smallest member identified, consists of only 99 amino acids (aa). It is hypothesized to be either a homologous protein or a partial fragment of the CYP, necessitating further investigation to elucidate the structure and function of CYP3A19. These genes are mapped to chromosomes 1–7 and 9 of *D. magna* (Table 2, Figure 1). Clan 4, the largest subgroup of CYPs in *D. magna*, is characterized by gene clusters distributed across all five chromosomes of the species. Gene clustering, indicative of duplication events, is most pronounced in Clan 4, which is predominantly located on the LG7 chromosome linkage group in *D. magna* (Figure 1). This suggests that extensive duplication events have occurred within the CYP4 clan genes on these chromosomes.

Based on sequence homology and evolutionary relationships among seven species, including *D. magna* and *D. pulex*, CYP genes in *D. magna* are predominantly organized into four clans (namely CYP 2–4 and the mitochondrial clan), seven families, and eight subfamilies, with each CYP gene associated with distinct functions (Table 1 and Table 2). CYP genes have been more conserved throughout the evolutionary transition from invertebrates to vertebrates [57]. Additionally, it has been demonstrated that only two-thirds of the P450 gene family is shared between *D. magna* and *D. pulex* [32,58]. The clan CYP1 is absent in invertebrates [25], including *D. magna*. CYP1 genes have been employed as environmental biomarkers for monitoring aquatic pollution [22]. For instance, CYP1A mRNA expression was significantly elevated in the liver of juvenile *Sebastes schlegelii* following exposure to 100 mg/kg oil for 12 h [30]. In marine medaka (*Oryzias melastigma*), the transcriptional level of CYP1 has been significantly upregulated in response to β-NF and B[α]P [22]. In channel catfish (*Ictalurus punctatus*), CYP1B1 exhibited high levels of expression in the gills, liver, blood, and gonads following exposure to B[a]P (benzo [a]pyrene) [30]. This diversity may be attributable to the variability and extent of evolutionary divergence of CYPs among species and their involvement in diverse physiological processes. The phylogenetic tree also indicated a higher similarity between CYPs sequences of *D. magna* and *D. pulex* than other organisms; as *D. magna* and *D. pulex* are from the same *Daphnia*, they would be expected to have much more similarity than species (Figure 2). The *D. magna* CYP2C15, together with human CYP2C8, CYP2C18, CYP2C9, and CYP2C19, were classified as family 2 homologous proteins and clustered within the Clan 2 clade in the phylogenetic analysis (Figure 2). These results suggest that CYP gene clusters across species may have evolved along divergent evolutionary pathways.

CYPs constitute a biologically ubiquitous mega-protein family characterized by diverse protein structures and functions. Although the amino acid sequences of different families vary, the domain generally comprises a folded structure at the N-terminal end and a helical bundle at the C-terminal end [23,52]. The functional structural domains of CYPs are highly conserved, particularly the electron-accepting heme-binding site (Figure 3A–C), which includes an invariant cysteine residue forming a thiolate ionic bond with iron, as well as helix C, which forms an electron zipper with heme, and helix K, which stabilizes motifs 1, 3, 6, and 7, conserved motifs shared by 16 CYPs in *D. magna* (Figure 3C). The 17 CYPs identified in *D. magna* possess the CYP4 domain. By analyzing the positions of the motifs within the CYP genes sequences, we discovered that these five motifs (motif 1, 3, 7, 11, and 13) are situated within the CYP4 domain, suggesting that these sites are closely related to the biological functions of the kinases and are thus evolutionarily conserved. The CYP genes belonging to the same subfamily were similar in gene structure and conserved motifs, indicating that the genes of the same subfamily may perform similar functions in cellular activities of *D. magna* (Figure 4). There were obvious differences in gene structure and conserved motifs among different subfamilies, indicating that different subfamilies may play different roles in the physiological activities of *D. magna* (Figure 4).

Metal oxide nanoparticles can induce oxidative stress and disrupt cellular homeostasis in exposed organisms, leading to the upregulation of detoxification pathways, particularly those involving CYP enzymes [59]. RNA-seq data have facilitated the identification of numerous CYP genes in species such as the Javanese medaka (*Oryzias javanicus*) [28], zebrafish (*Danio rerio*) [27] and Spotted sea bass (*Lateolabrax maculatus*) [29]. Our results demonstrate significant modulation of multiple CYP genes in *D. magna* following exposure to nCeO_2_ (Figure 5A,B). Specifically, certain genes were upregulated, indicating an active response to the oxidative stress induced by metal oxide nanoparticles. Notably, the upregulation of specific CYP genes, such as CYP4 and CYP370, suggests their involvement in the organism’s detoxification processes. CYP2 and CYP4 have been identified as playing significant roles in the detoxification processes of invertebrates under xenobiotic stress [60]. Our findings demonstrate that exposure to nCeO_2_ induces the expression of several CYP genes in *D. magna*. Specifically, genes such as CYP18A1, CYP2K4, and CYP306a1, which belong to the Clan 2, exhibited downregulation at 48 h exposed to nCeO_2_ (Figure 5B). This downregulation at various exposure times indicates a robust detoxification response to nCeO_2_, suggesting that these genes play a critical role in metabolizing and mitigating the toxic effects of nCeO_2_. The observed dysregulation patterns align with previous studies where various CYP genes in aquatic organisms were induced by exposure to metal oxide nanoparticles, reinforcing the potential of these genes as biomarkers for environmental monitoring.

Our findings demonstrate that exposure to nCeO_2_ induces the expression of several CYP genes in *D. magna* (Figure 5A,B). CYP1A was significantly upregulated in response to gold nanoparticles [59], B[a]P [30], and oxybenzone [38] at earlier exposure times, underscoring its broad-spectrum role in detoxification processes. Cyp306A1 and Cyp307A1, conserved Halloween genes, play crucial roles in the ecdysteroid biosynthetic pathway, which is associated with molting and metamorphosis in invertebrates [22,32]. Cyp17a1, which efficiently converts progesterone and pregnenolone to androstenedione and dehydroepiandrosterone in Nile tilapia (*Oreochromis niloticus*), is believed to play essential roles in steroidogenesis [61]. Higher expression of CYP3C1 was detected in zebrafish brains at both 48 and 120 h of embryonic development, suggesting that CYP3C1 is associated with early brain development [34]. The observed upregulation of specific CYP genes, such as CYP4C1 and CYP4c3, in response to nCeO_2_ exposure is analogous to the increased expression of CYP genes in response to other stressors, such as toxicants [62] and pollutants [63], in various aquatic organisms. The CYPs are evolutionarily conserved, indicating that *D. magna* CYPs could be implicated in the stress response to metal oxide nanoparticles by regulating critical cell processes, such as redox balance [56], apoptosis [26], and autophagy [36].

## 4. Materials and Methods

### 4.1. Experimental Materials and Toxicity Treatment

*D. magna* specimens were obtained from the Key Laboratory of Hydrobiology at Dalian Ocean University, Liaoning Province, China. The adults used in the experiments were synchronously released from a laboratory-cultured clonal line, which was initiated with a single parthenogenetic female. Experiments utilized three to six brooding offspring. Neonates or adults selected for experimentation were cultured in dechlorinated water within 1000 mL glass beakers and were maintained in an illumination incubator (Thermo Fisher, Dreieich, Germany). Cultures were maintained under an 18 h: 6 h light/dark photoperiod and at a temperature of 22 ± 2 °C. The light intensity parameter of the experimental animal culture was set to 2000 lux. The total hardness, alkalinity, dissolved oxygen, and pH of culture water were 7.29 ± 0.1 mmol/L, 12.48 ± 0.4 mmol/L, 7.96 ± 0.3 mg/L, and 7.8 ± 0.2, respectively. *D. magna* fed 2.5 × 10^6^ cells of the single-celled green algae *Tetradesmus obliquus* daily.

Cerium (IV) oxide nanoparticles (CAS No: 1306-38-3, MW: 172.11 g/mol, nominal size < 50 nm, purity 99.95%, manufacturer’s data) was bought from Sigma-Aldrich (St. Louis, MO, USA). The dry nCeO_2_ powder was stored in the dark at 24 °C, and stock suspensions were freshly prepared prior to each use. For whole-animal exposure treatments, stock suspensions were prepared by dispersing nCeO_2_ in 500 mL of dechlorinated water, followed by sonication using a KQ3200DE Sonic Dismembrator (Kunshan Ultrasound Instrument Co., Ltd. Suzhou, China) at full power for 30 min to achieve a final concentration of 1 mg/mL [11].

Experimental groups were established, including a blank control and nCeO_2_ treatment groups at concentrations of 10, 50, and 150 mg/L for 24 h and 48 h exposures. Each group was replicated four times to mitigate the impact of variability on the results. Approximately 50 *D. magna* were collected from each group and transferred to 1.5 mL EP tubes for RNA extraction. Adult *D. magna* specimens, with an average length of 2.67 ± 0.05 mm, were utilized for the toxicity exposure tests.

### 4.2. Identification of CYPs in Daphnia magna

*D. magna* genome sequences (assembly ASM2063170v1.1: *Daphnia magna*—Assembly—NCBI) were obtained from the National Center for Biotechnology Information (NCBI). To seek for candidate CYPs, first, the corresponding amino acid sequences were obtained using the BLAST search. Moreover, the HMM profile for CYP domains (PF00067) was obtained from the Pfam protein family database and used in Tbtools to search for putative *D. magna* CYPs. The existence of conserved CYP domains was also confirmed by using Tbtools and the Conserved Domain Database (CDD) from NCBI. The basic protein characteristics, such as the sequence length, molecular weight, and isoelectric point (pI) were predicted by using Compute pI/MW tool from the ExPASy server. The cellular compartmentalization of CYP450 proteins was predicted with WOLF PSORT. Finally, the localization of CYPs on chromosomes was determined using TBtools, with the help of genomic data from the *D. magna* database.

### 4.3. Transcriptomic Analysis of CYPs in Daphnia magna Exposed to nCeO_2_

The transcriptome sequencing data for *D. magna* have been deposited in the NCBI SRA database (PRJNA983527). This dataset provides a detailed analysis of gene expression differences in *D. magna* exposed to 50 mg/L of nCeO_2_ for 48 h. The data presented in this study are openly available in the NCBI SRA database (PRJNA983527) accessed on 2 July 2025.

### 4.4. Chromosomal Distribution

The chromosomal distribution of most CYPs in *D. magna* was mapped using TBtools based on location information obtained from the *D. magna* genome database.

### 4.5. Phylogenetic Tree

Multiple sequence alignments of predicted CYP protein sequences were performed using ClustalW and Weblogo with default settings. A phylogenetic analysis was conducted using MEGA 11 software (Pennsylvania State University, Philadelphia, PA, USA), employing the neighbor-joining method, and based on CYP amino acid sequences from eight species: *D. magna*, *Penaeus chinensis*, *Drosophila innubila*, *Daphnia pulex*, *Danio rerio*, *Mus musculus*, *Homo sapiens*, and *Eriocheir sinensis*. The robustness of the phylogenetic tree was assessed using 1000 bootstrap replicates, and the final tree was refined and visualized with the Evolview platform (https://evolgenius.info/evolview-v2/) accessed on 30 September 2024.

### 4.6. Structure and Motifs of CYP Genes in Daphnia magna

The gene structure of CYPs was analyzed using TBtools by examining their coding sequences. To identify the conserved motifs within the CYP protein family in *D. magna*, the MEME 5.5.4 tool (https://meme-suite.org/meme/tools/meme) accessed on 9 July 2024 was employed, setting the maximum number of motifs to 15, with all other parameters kept at default settings. The conserved motifs identified were subsequently visualized using TBtools 2.003 (TB-Tools, Ningbo, China) based on the output MAST.XML files generated using the MEME suite.

### 4.7. Quantitative Real-Time PCR (qPCR) Analysis

RNA was isolated from each *D. magna* sample using TRIzol reagent (Sangon Biotech, Shanghai, China) and purified with phenol–chloroform and ethanol precipitation. The concentration and quality of RNA were measured by NanoDrop 2000 spectrophotometer (NanoDrop Technologies, Wilmington, NC, USA). Reverse transcription PCR was performed using the Evo M-MLV RT Kit (Accurate Biotechnology Co., Ltd., Changsha, China) following the manufacturer’s protocol. The qPCR analysis was conducted using an Applied Biosystems™ 7500 RT-PCR System (Agilent Technologies, Santa Clara, CA, USA) and a SYBR^®^ Green Premix Pro Taq HS qPCR Kit (Rox Plus) obtained from Accurate Biotechnology Co., Ltd. Specific CYP gene primers were designed using the NCBI Primer-BLAST tool (Table 3). *D. magna* β-actin was used as the internal control gene [64]. The expression levels of the CYP genes were then compared to the control group using the 2^−∆∆Ct^ method. All reactions were performed in three biological replicates and technical replicates for each biological replicate. Statistical analysis of CYP gene expression between the control and nCeO_2_-treated groups was conducted using Excel 2016 (Microsoft, Redmond, WA, USA) for the *t*-test and GraphPad 8.0.2 (GraphPad Software, San Diego, CA, USA) for the one-way ANOVA. A *p*-value of less than 0.05 was considered to indicate a statistically significant difference between the treated and control groups.

## 5. Conclusions

Comprehensive genome-wide identification and expression profiling of the CYPs superfamily in *D. magna* exposed to nCeO_2_ has yielded critical insights into the adaptive responses of these genes and their potential utility as biomarkers in environmental surveillance. The twenty-six identified CYP genes in *D. magna* are unevenly distributed across seven distinct subfamilies. CYP enzymes are implicated in diverse cellular processes such as maintaining redox homeostasis, regulating apoptosis, and inducing autophagy, playing vital roles in growth, development, and stress resilience in *D. magna*. nCeO_2_ exposure in *D. magna* profoundly altered the expression of several CYP genes, such as CYP4C1.3, CYP18a1, CYP4C1.1, and CYP4c3.9, which may consequently perturb critical CYP-driven physiological functions. This study establishes a foundation for further investigations into the molecular mechanisms governing xenobiotic detoxification and stress adaptation in aquatic invertebrates.

## Figures and Tables

**Table 1:** Annotated molecular function of Cytochrome P450s in *Daphnia magna*.

Clan	Gene Name	Gene ID	Molecular Function
Clan 2	CYP2C15	LOC116918329	steroid hydroxylase activity
	CYP2K4	LOC116923437	steroid hydroxylase activity
	CYP18a1	LOC116919046	steroid hydroxylase activity
	CYP306a1	LOC116918853	steroid hydroxylase activity; steroid hydroxylase activity
Clan 3	CYP3A19	LOC116928063	heme binding, iron ion binding, monooxygenase activity, oxidoreductase activity, acting on paired donors, with incorporation or reduction in molecular oxygen
Clan 4	CYP4C1.1	LOC116927068	heme binding, iron ion binding, monooxygenase activity, oxidoreductase activity, acting on paired donors, with incorporation or reduction in molecular oxygen
	CYP4C1.2	LOC116926713	heme binding, iron ion binding, methyltransferase activity, monooxygenase activity, oxidoreductase activity, acting on paired donors, with incorporation or reduction in molecular oxygen,
	CYP4C1.3	LOC116926407	iron ion binding; oxidoreductase activity, acting on paired donors, with incorporation or reduction in molecular oxygen; heme binding; oxidation-reduction process
	CYP4C1.4	LOC116926406	iron ion binding; oxidoreductase activity, acting on paired donors, with incorporation or reduction in molecular oxygen; heme binding; oxidation-reduction process; oxidation-reduction process
	CYP4C1.5	LOC116926459	heme binding, iron ion binding, monooxygenase activity, oxidoreductase activity, acting on paired donors, with incorporation or reduction in molecular oxygen
	CYP4C1.6	LOC116928122	iron ion binding; oxidoreductase activity, acting on paired donors, with incorporation or reduction in molecular oxygen; heme binding; oxidation-reduction process
	CYP4C1.7	LOC116927585	heme binding, iron ion binding, monooxygenase activity, oxidoreductase activity, acting on paired donors, with incorporation or reduction in molecular oxygen
	CYP4C1.8	LOC116928121	heme binding, iron ion binding, monooxygenase activity, oxidoreductase activity, acting on paired donors, with incorporation or reduction in molecular oxygen
	CYP4C1.9	LOC116927935	heme binding, iron ion binding, monooxygenase activity, oxidoreductase activity, acting on paired donors, with incorporation or reduction in molecular oxygen
	CYP4C1.10	LOC116930969	iron ion binding; oxidoreductase activity, acting on paired donors, with incorporation or reduction in molecular oxygen; heme binding; oxidation-reduction process; oxidation-reduction process
	CYP4c3.1	LOC116929157	heme binding, iron ion binding, monooxygenase activity, oxidoreductase activity, acting on paired donors, with incorporation or reduction in molecular oxygen
	CYP4c3.2	LOC116922314	heme binding, iron ion binding, monooxygenase activity, oxidoreductase activity, acting on paired donors, with incorporation or reduction in molecular oxygen
	CYP4c3.3	LOC116925875	iron ion binding; oxidoreductase activity, acting on paired donors, with incorporation or reduction in molecular oxygen; heme binding; oxidation-reduction process
	CYP4c3.4	LOC116926314	heme binding, iron ion binding, monooxygenase activity, oxidoreductase activity, acting on paired donors, with incorporation or reduction in molecular oxygen
	CYP4c3.5	LOC116934506	heme binding, iron ion binding, monooxygenase activity, oxidoreductase activity, acting on paired donors, with incorporation or reduction in molecular oxygen
	CYP4c3.6	LOC116934248	heme binding, iron ion binding, monooxygenase activity, oxidoreductase activity, acting on paired donors, with incorporation or reduction in molecular oxygen
	CYP4c3.7	LOC116926402	iron ion binding; oxidoreductase activity, acting on paired donors, with incorporation or reduction in molecular oxygen; heme binding; oxidation-reduction process
	CYP4c3.8	LOC116926463	heme binding, iron ion binding, monooxygenase activity, oxidoreductase activity, acting on paired donors, with incorporation or reduction in molecular oxygen
	CYP4c3.9	LOC116926461	heme binding, iron ion binding, monooxygenase activity, oxidoreductase activity, acting on paired donors, with incorporation or reduction in molecular oxygen
Mitochondrial clan	CYP302a1	LOC116927720	iron ion binding; mitochondrion; ecdysone biosynthetic process; dorsal closure; central nervous system development; midgut development; head involution; chitin-based embryonic cuticle biosynthetic process; electron transfer activity; oxidoreductase activity, acting on paired donors, with incorporation or reduction in molecular oxygen; heme binding; ecdysteroid 22-hydroxylase activity; oxidation-reduction process
	CYP315a1	LOC116926680	iron ion binding; mitochondrion; ecdysone biosynthetic process; dorsal closure; central nervous system development; midgut development; motor neuron axon guidance; head involution; oxidoreductase activity, acting on paired donors, with incorporation or reduction in molecular oxygen; heme binding; ecdysteroid 2-hydroxylase activity; oxidation-reduction process

**Table 2:** Physicochemical properties of Cytochrome P450s in *Daphnia magna*.

Gene Name	Chromosome Location	Length (aa)	Isoelectric Points	Molecular Weight/Da	Subcellular Localizations	Exon Count	Gravy
CYP18a1	LG1	551	8.35	63,170.02	extracellular	6	−0.122
CYP4C1.9	LG7	506	8.72	58,694.89	plasma membrane	18	−0.17
CYP2C15	LG3	507	9.19	58,789.94	endoplasmic reticulum	9	−0.181
CYP4C1.6	LG7	504	6.55	58,039.1	plasma membrane	12	−0.119
CYP4c3.1	Unknown	514	8.23	58,912.73	mitochondrion	13	−0.13
CYP4C1.4	LG7	444	8.43	50,380.05	mitochondrion	14	−0.15
CYP4c3.4	LG7	528	7.17	60,877.57	endoplasmic reticulum	13	−0.141
CYP4c3.7	LG7	526	6.3	59,991.96	plasma membrane	14	−0.18
CYP306a1	LG1	585	8.45	66,441.76	plasma membrane	9	−0.159
CYP4C1.1	LG7	520	6.22	60,095.44	plasma membrane	16	−0.114
CYP4C1.2	LG7	509	6.75	58,800.96	endoplasmic reticulum	11	−0.173
CYP4c3.5	LG7	490	7.87	55,885.2	plasma membrane	15	−0.097
CYP4C1.10	LG9	527	7.61	60,180.6	endoplasmic reticulum	12	−0.054
CYP3A19	LG7	99	9.16	11,683.6	extracellular	4	−0.363
CYP4c3.9	LG7	528	6.1	60,656.91	endoplasmic reticulum	11	−0.15
CYP4C1.3	LG7	509	5.93	58,157.05	plasma membrane	12	−0.053
CYP4c3.8	LG7	534	8.58	62,191.37	endoplasmic reticulum	13	−0.159
CYP4c3.3	LG6	514	6.9	59,866.09	endoplasmic reticulum	11	−0.263
CYP2K4	LG5	503	7.17	58,164.88	endoplasmic reticulum	9	−0.191
CYP4c3.6	LG7	520	6.44	58,821.43	plasma membrane	14	0.058
CYP4C1.8	LG7	507	7.6	58,335.33	plasma membrane	11	−0.173
CYP4C1.7	LG7	513	8.43	58,957.01	endoplasmic reticulum	13	−0.209
CYP4C1.5	LG7	533	6.65	60,999.32	plasma membrane	11	−0.135
CYP4c3.2	LG5	256	5.98	28,663.33	extracellular	6	−0.001
CYP315a1	LG7	491	8.77	56,522.29	mitochondrion	8	−0.229
CYP302a1	LG7	532	9.43	60,986.62	mitochondrion	8	−0.269

**Table 3:** Primers sequence for qPCR.

Gene ID	Description	Gene	Forward Primer (5′-3′) Reverse Primer (3′-5′)
	Beta-actin	Beta-actin	CCCCATTTATGAAGGTTACGC CCTTGATGTCACGGACGATTT
116919046	cytochrome P450 18a1	CYP18a1	TCACCATACCGAAAGGCACC ACGCCGAACGGAATGAAGTA
116927935	cytochrome P450 4C1.9	CYP4C1.9	ATATGCCCTCCACCACAACG TCCAATGCAGTTCCTCGGTC
116918329	cytochrome P450 2C15	CYP2C15	TCATACTCACAGCGAACGCA ATCATGCCAAAGGGCAGTGT
116928122	cytochrome P450 4C1.6	CYP4C1.6	TTCGGCAAGTGGACGACATC CCTCGACATCGGAAGGAGAC
116929157	cytochrome P450 4c3.1	CYP4c3.1	TGGCTTCGATTGGATTGGCT ACCAATTCACGGCGATGTCT
116926406	cytochrome P450 4C1.4	CYP4C1.4	ATCACCAGAATTTATGGAGGTAAGT CCGGGACTAATAAGAAGCCCT
116926314	cytochrome P450 4c3.4	CYP4c3.4	TACCTGATGGCGAAGCATCC GGATGCCAGGGTACAACCTC
116926402	cytochrome P450 4c3.7	CYP4c3.7	CAGAGAACAGCATCGGTCGT CATAGCTGGATCAGAGGCGG
116918853	cytochrome P450 306a1	CYP306a1	TTTGCCCAGTTACCCAGTTGT TGCAACCACCATACGGCGA
116927068	cytochrome P450 4C1.1	CYP4C1.1	TAATAACGCGGGGCTGAGTG TCGCTGATGTTGTCGTGTCA
116926713	cytochrome P450 4C1.2	CYP4C1.2	CATCAAGCGCCGTGTTAGTG ACGGAACGAAGGCAAATGGA
116934506	cytochrome P450 4c3.5	CYP4c3.5	GTCGGTGCTGTGGGATTGTT CGTATCTTCCCGTCCACGTT
116930969	cytochrome P450 4C1.10	CYP4C1.10	TGGAAACCCAAAAGGACGGT ACAGCTTGAGGGTATTCGCC
116928063	cytochrome P450 3A19	CYP3A19	ATGCCGGCGTATGCTCTC TTTCAGCGCACCATCTTTCG
116926461	cytochrome P450 4c3.9	CYP4c3.9	CCGGAGAACAGTATCGGACG TCATGGGCTGTGAGGAATCG
116926407	cytochrome P450 4C1.3	CYP4C1.3	TCTTGATGATATGTGCGCGTC GCGCGTTTATTCTTGTCCCC
116926463	cytochrome P450 4c3.8	CYP4c3.8	TAACACCGGCATTCCACGTT ATGTCAAGGGCGTGTTCCAT
116925875	cytochrome P450 4c3.3	CYP4c3.3	CTGACTCCGGCCTTCCATTT ATGTCGAGGGTGCATCGTTT
116923437	cytochrome P450 2K4	CYP2K4	CTGAAGCTCGCCAAATGGTC CTCGCTAAACTTGTCCGCCT
116934248	cytochrome P450 4c3.6	CYP4c3.6	GGTCCCAGGT TCTACTTGCC TAGCAATGCGAGCCAAGGAA
116928121	cytochrome P450 4C1.8	CYP4C1.8	AATTTGGCCGCATCTACCGA TTCCACTTAGCCCCTGTTGC
116927585	cytochrome P450 4C1.7	CYP4C1.7	AGGGTGGAGTGGAGCTTAGT TGATGCCCTGGATTCGTAGC
116926459	cytochrome P450 4C1.5	CYP4C1.5	CAAGCGTCGGAGAATGGAGA AGAAACCAACTCATCGCCGT
116922314	cytochrome P450 4c3.2	CYP4c3.2	AGAACATGCACGCTGAACAAC CTGGATCAGGGCTAACCTCC
116926680	cytochrome P450 315a1	CYP315a1	AATGGAACGACCACCACCAT TGCACTTGGAACGTGCAATC
16927720	cytochrome P450 302a1	CYP302a1	TTGGGACGGTCTCTGTGTTG GGTAGTGGTTTAGGGCAAGGT

## Data Availability

The data supporting this study’s findings are available from the corresponding authors upon reasonable request.
